# Carcinoid tumor of the minor papilla in complete pancreas divisum presenting as recurrent abdominal pain

**DOI:** 10.1186/1471-230X-10-17

**Published:** 2010-02-12

**Authors:** Yong Gil Kim, Tae Nyeun Kim, Kyeong Ok Kim

**Affiliations:** 1 Department of Internal Medicine, Yeungnam University College of Medicine, Daegu, South Korea

## Abstract

**Background:**

Tumors of the minor papilla of the duodenum are extremely rare, and they are mostly neuroendocrine tumors, such as somatostatinomas and carcinoid tumors. However, true incidence of carcinoid tumors in minor papilla might be much higher, because patients with minor papillary tumors usually remain asymptomatic. We report a very unusual case of carcinoid tumor in a patient with complete pancreas divisum with a review of the literature.

**Case presentation:**

A 56-year-old female patient was referred for evaluation of pancreatic duct dilatation noted on abdominal ultrasonography and computerized tomography. She complained of intermittent epigastric pain for 6 months. A MRCP and ERCP revealed complete pancreas divisum with dilatation of the main pancreatic duct. On duodenoscopy, a small, yellows, subepithelial nodule was visualized at the minor papilla; biopsy of this lesion revealed a carcinoid tumor. She underwent a pylorus-preserving pancreaticoduodenectomy. The histologic evaluation showed a single nodule, 1 cm in diameter, in the submucosa with duodenal and vascular invasion and metastasis to the regional lymph nodes.

**Conclusion:**

Although the size of the carcinoid tumor was small and the tumor was hormonally inactive, the concomitant pancreas divisum led to an early diagnosis, the tumor had aggressive behavior. Carcinoid tumors of the minor papilla should be included in the differential diagnosis of recurrent abdominal pain or pancreatitis of unknown cause.

## Background

Tumors of the minor papilla of the duodenum are very rare; the majority of tumors of the minor papilla of the duodenum are neuroendocrine tumors (NETs), such as somatostatinomas and carcinoid tumors [1-7]. Carcinoid tumors arise from enterochromaffin cells, and gastrointestinal carcinoids are usually located in the appendix, ileum, stomach, and rectum. Carcinoids of the minor papilla are extremely rare; only 8 cases have been reported in the literature [1-8].

Pancreas divisum is the most common congenital anomaly of the pancreas, resulting from failure of fusion between the dorsal and ventral pancreatic ducts. Most exocrine secretions of the pancreas drain through the dorsal pancreatic duct and the minor papilla in the pancreas divisum. Patients with pancreas divisum usually have no clinical symptoms, but sometimes have recurrent abdominal pain or pancreatitis when disturbances in drainage of pancreatic secretion through the minor papilla occur.

We report a very unusual case of a carcinoid tumor of the minor papilla arising in a patient with complete pancreas divisum who presented with recurrent abdominal pain.

## Case persentation

A 56-year-old female was referred to our department for evaluation of dilatation of the main pancreatic duct noted on abdominal ultrasonography and CT scan. She complained of intermittent epigastric pain 6 months prior to the referral. She did not drink alcohol or take any medications, and had no significant medical history. She denied sweating, diarrhea, or facial flushing.

Laboratory data on admission were as follows: total bilirubin, 0.47 mg/dL; AST, 41 U/L; ALT, 33 U/L; ALP, 161 U/L; GGT, 32 U/L; and amylase, 147 U/L. The complete blood count, urine analysis, and serum electrolytes were within normal limits. The CA 19-9 was 7.99 U/ml and the 5-HIAA was 21.44 mg/day.

**Figure 1:**
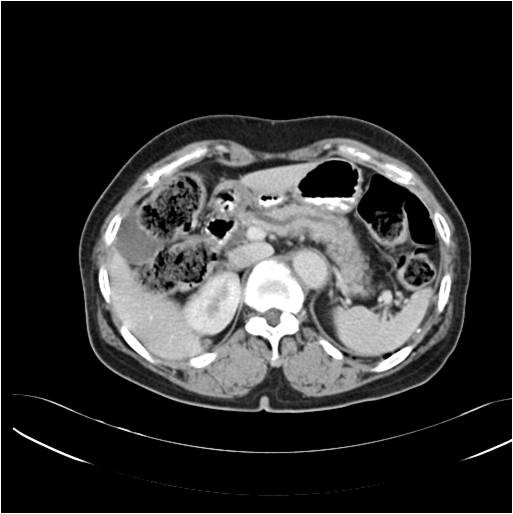
**Abdominal CT finding**. The pancreatic duct was diffusely dilated without a definite obstructive mass lesion.

**Figure 2:**
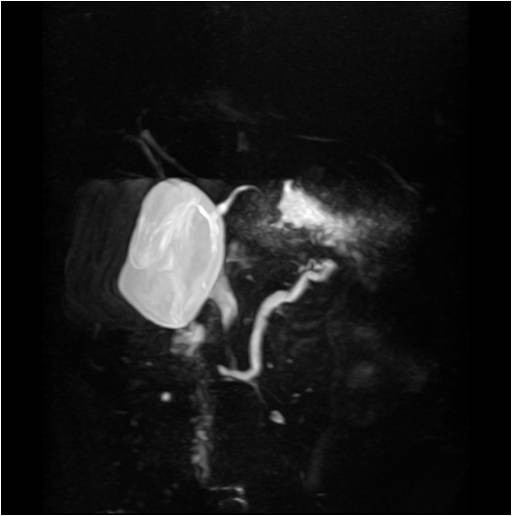
**Magnetic resonance cholangiopancreatographic finding**. The dorsal pancreatic duct was slightly enlarged with stenosis at the minor papilla. Furthermore, it crossed over the common bile duct. The ventral duct appeared to be normal and there was no communication between the dorsal and ventral ducts, indicating pancreas divisum.

**Figure 3:**
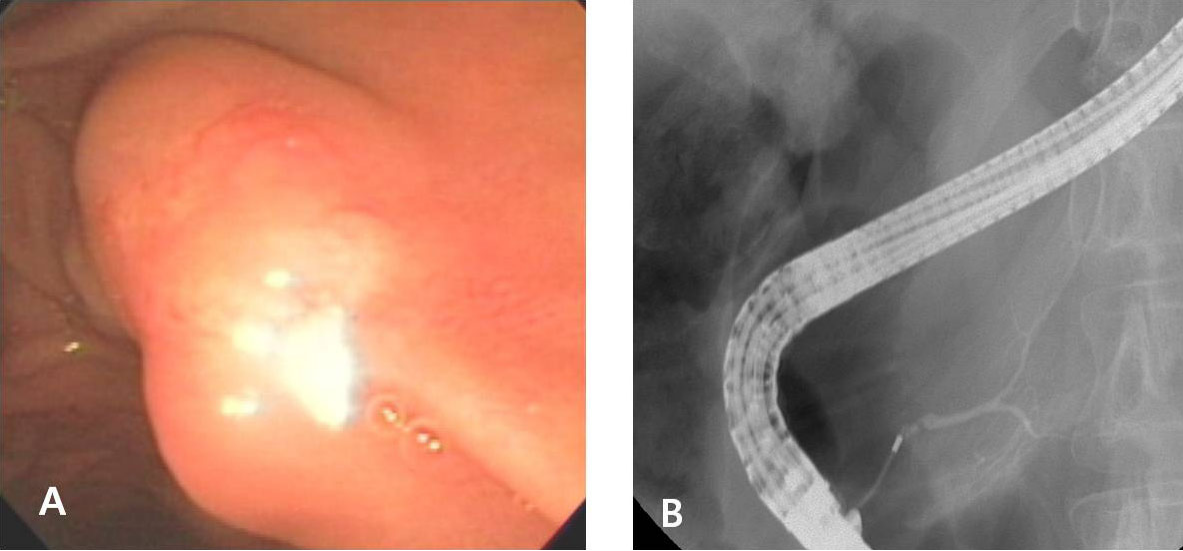
**ERCP finding**. **A**, Duodenoscopic examination revealed 1 cm sized yellowish bulging nodular lesion with intact mucosal surface at minor papilla. **B**, Injection of contrast through major papilla revealed short and slender ventral duct confined to pancreas head with no visualization of ducts in body and tail. The ventral duct was gradually diminishing in caliber and arborizing.

**Figure 4:**
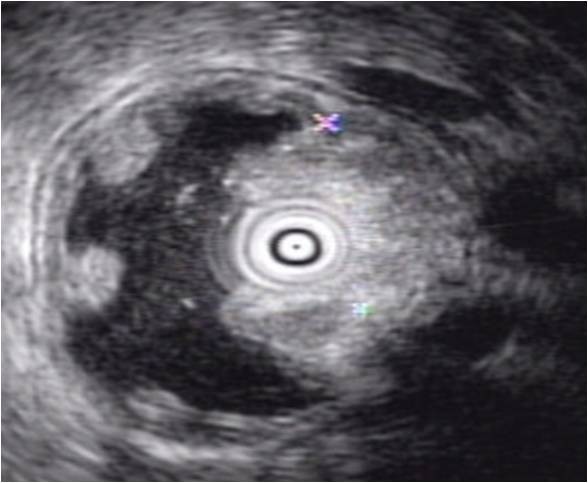
**Endoscopic ultrasonography finding**. There was 1.3 cm sized ill defined hypoechoic mass in submucosal layer at minor papilla.

On the CT scan, the pancreatic duct was diffusely dilated without a definite mass and the common bile duct was slightly dilated (Figure 1). Magnetic resonance cholagiopancreatography (MRCP) showed a dilated dorsal pancreatic duct crossing over the common bile duct with stenosis at the minor papailla. The ventral duct appeared to be normal and there was no communication between the dorsal and ventral ducts, indicating pancreas divisum (Figure 2). The common bile duct was slightly dilated, and narrowing of the distal common bile duct (CBD) was noted without passage disturbance. A duodenoscopic examination revealed a 1 cm yellow, bulging, nodular lesion with an intact mucosal surface in the minor papilla. The orifice of the minor papilla was not visible due to the nodule and cannulation was not possible (Figure 3A). On endoscopic retrograde cholangiopancreatography (ERCP), the major papilla appeared to be normal. Injection of contrast through the major papilla revealed a short and slender ventral duct confined to the pancreas head with no visualization of ducts in the body and tail. The ventral duct gradually diminished in caliber and was arborizing (Figure 3B). Endoscopic ultrasonography (EUS) showed a 1.3 cm ill-defined hypoechoic mass in the submucosal layer of the minor papilla (Figure 4). Multiple deep biopsies of this nodule revealed a carcinoid tumor.

**Figure 5:**
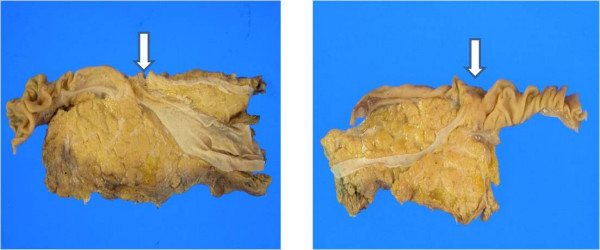
**Gross findings of resected specimen**. The resected specimen was composed of duodenum, pancreatic head with a part of pancreatic body and common bile duct. There was 1.2 × 1.0 cm sized submucosal lesion at minor papilla (arrow). There was no communication between common bile duct (CBD) and pancreatic duct (PD). Furthermore, dorsal pancreatic duct only connected with duct of Santorini and without any branch to major papilla. These findings were appropriate to pancreas divisum.

Under the diagnosis of a carcinoid tumor of the minor papilla in complete pancreas divisum, the patient underwent a pylorus-preserving pancreaticoduodenectomy. The resected specimen consisted of duodenum, pancreas, and CBD. There was a 1.2 × 1.0 cm submucosal lesion of the minor papilla (Figure 5).

**Figure 6:**
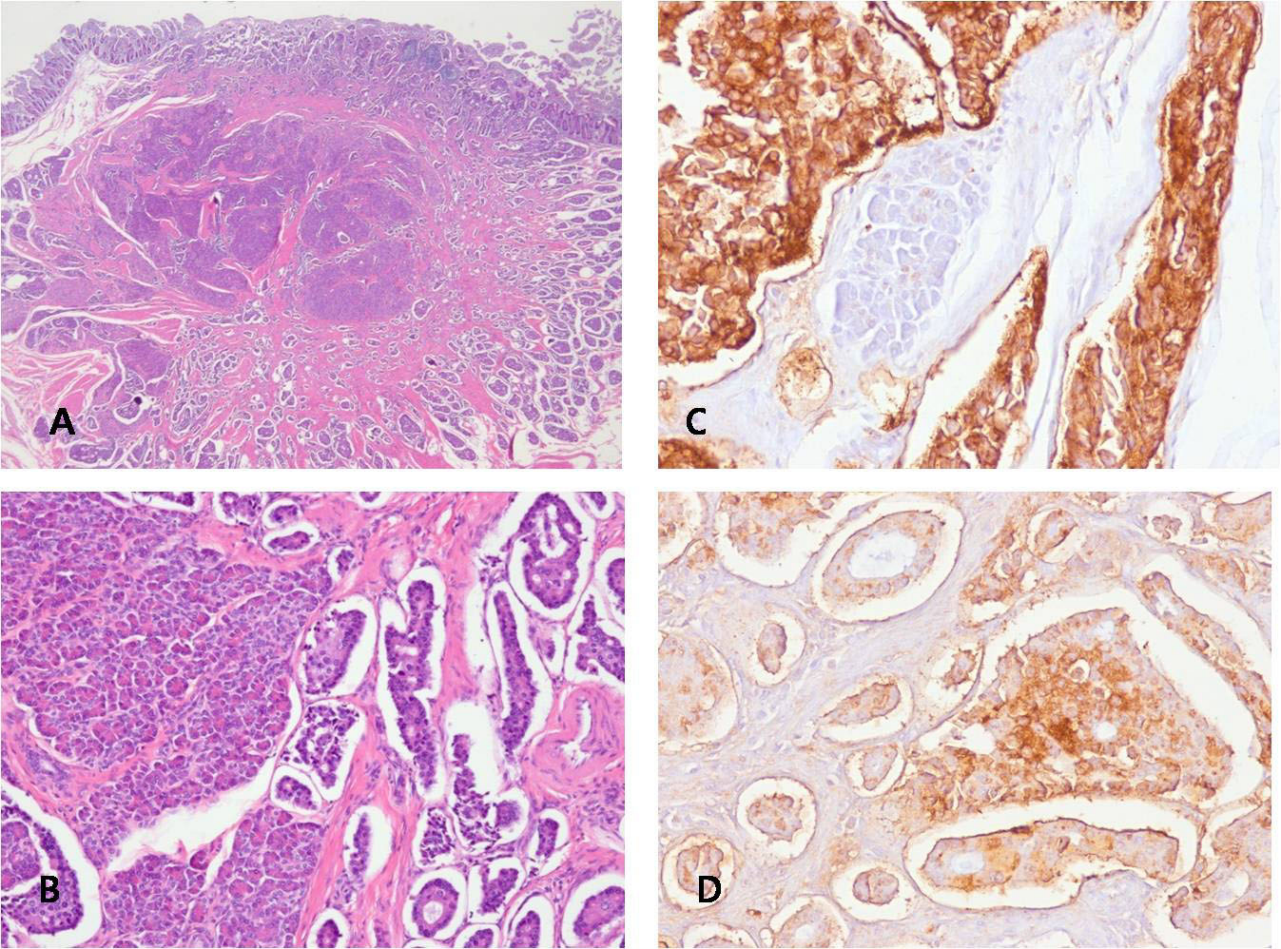
**Microscopic examination**. **A, B**, Heterotopic pancreas in the duodenal submucosa and muscularis propria around the minor papilla was noted. There were uniform and round-shaped cells in or around the heterotopic pancreas and they were arranged in a solid nest or tubular pattern. These findings were suggestive of a carcinoid tumor. There was vascular invasion, but no perineural invasion was noted. (H&E stain A: ×10, B: ×100) **C, D**, According to immunohistochemical staining, the tumor cells were immunoreactive with neuroendocrine markers, such as synaptophysin and chromogranin.(C:Synaptophysin stain ×100; D: Chromogranin stain ×100)

On the microscopic examination, uniform and round-shaped cells were visualized in or around the heterotopic pancreas in the minor papilla, arranged in a solid nest or tubular pattern, suggesting a carcinoid tumor. There was vascular invasion, but no perineural invasion was noted (Figures 6A, B). Among the four resected regional lymph nodes, 2 metastatic nodes were detected. The tumor cells showed immunoreactivity to neuroendocrine markers, such as synaptophysin and chromogranin (Figures 6C, D) The patient has been without abdominal pain for 1 year after surgery.

## Discussion

The term carcinoid has been replaced with NET based on the development of diagnostic tools and immunochemistry. Recently, NETs have been reclassified within the spectrum of gastroenteropancreatic-neuroendocrine tumours (GEP-NETs) [9]. However, the term "classical carcinoid" is used synonymously with the term serotonin-producing GEP-NETs [10]. A classical carcinoid tumor is usually still referred to as a carcinoid tumor. The incidence of gastrointestinal carcinoids is 1.6-2.0 cases per 100, 000 persons per year [11], but the true prevalence may be higher [12]

Although more than 70 cases involving carcinoid tumors of the major papilla have been reported [13], only 8 cases of carcinoid tumors involving the minor papilla have been reported [1-8]. Carcinoids and endocrine cell micronests (ECMs) in the minor papilla occur more frequently than generally thought. In a single surgical specimens and autopsy study, the incidence of carcinoids and neoplastic ECMs of the minor papilla could reach 10%. Furthermore, carcinoids in the minor papilla are twice as common as carcinoids of the major papilla, and neoplastic ECMs of the minor papilla are found five times as often [14]. This discrepancy may be explained by the fact that tumors of the major papilla are more likely to develop symptoms, such as jaundice or abdominal pain, due to ampullary obstruction, whereas patients with minor papillary tumors usually remain asymptomatic because there is no biliary or pancreatic obstruction.

Carcinoid tumors of the minor papilla of the duodenum are very difficult to diagnose because they are usually small in size and located at the submucosal area, and they are frequently asymptomatic [15] and rarely accompanied with endocrine manifestations [13]. In our case, although the size of the carcinoid tumor was small and the tumor was hormonally inactive, the concomitant pancreas divisum led to an early diagnosis because obstruction of the minor papilla due to the carcinoid tumor caused recurrent abdominal pain and resulted in dilatation of the main pancreatic duct. Of the eight reported cases of carcinoid tumors involving the minor papilla in the literature, five cases have been associated with pancreas divisum and presented with recurrent abdominal pain or pancreatitis [3-7].

The endoscopic features of carcinoid tumors are small, round, sessile, or polypoid lesions with a smooth surface. Carcinoid tumors usually have normal overlying mucosa and seldom ulcerate [16]. An EUS may be useful for the diagnosis of carcinoids and evaluation of regional lymph node metastasis, but CT or MRI scans are not helpful in most cases because of the small size. Carcinoid tumors usually appear as a homogenous, well-demarcated, and mildly hypoechoic or isoechoic mass that arises from the second layer on EUS. In addition, EUS can define the size, tumor invasion, and regional lymph node metastasis [16]. The pre-operative diagnosis of carcinoid tumors of the duodenal papillae is largely dependent upon endoscopic biopsy. However, a correct diagnosis is made in only 14% of the patients^13 ^because the tumors are usually submucosal in location [13]. As in our case, multiple deep biopsies are required to enhance the tissue diagnosis.

Though endoscopic resection may be an alternative to surgical resection [8,17], radical surgery is usually recommended as the treatment of ampullary carcinoid tumors [13,18] because carcinoid tumors of this area appear to have more aggressive biology with presence of metastasis in approximately 50% of the cases, irrespective of the size of the primary tumor or mitotic activity [13,19,20]. In our case, despite the tumor being 1 cm in diameter, the histologic examination revealed duodenal and vascular invasion and metastasis to the regional lymph nodes. In conclusion, carcinoid tumors of the minor papilla are very rarely found, but the true incidence might be much higher, and the tumors frequently have aggressive behavior. Carcinoid tumors of the minor papilla should be included in the differential diagnosis of recurrent abdominal pain or pancreatitis of unknown cause.

## Consent

Written informed consent was obtained from the patient for publication of this case report and accompanying images. A copy of the written consent is available for review by the Editor-in-Chief of this journal.

## Competing interests

The authors declare that they have no competing interests.

## Authors' contributions

All authors read and approved this manuscript. YGK analyzed and interpreted the patient's data. KOK collected the patient's data. TNK supervised this case report and followed up the patient.

## Pre-publication history

The pre-publication history for this paper can be accessed here:

http://www.biomedcentral.com/1471-230X/10/17/prepub
